# Dual Stem Cell Therapy Improves the Myocardial Recovery Post-Infarction through Reciprocal Modulation of Cell Functions

**DOI:** 10.3390/ijms22115631

**Published:** 2021-05-26

**Authors:** Sinziana Popescu, Mihai Bogdan Preda, Catalina Iolanda Marinescu, Maya Simionescu, Alexandrina Burlacu

**Affiliations:** Institute of Cellular Biology and Pathology “Nicolae Simionescu”, 8, B.P. Hasdeu Street, 050568 Bucharest, Romania; sinziana.popescu@icbp.ro (S.P.); bogdan.preda@icbp.ro (M.B.P.); catalina.marinescu@icbp.ro (C.I.M.); maya.simionescu@icbp.ro (M.S.)

**Keywords:** mesenchymal stromal cells, myocardial infarction, endothelial colony forming cells, proteomic profiling, dual stem cell therapy

## Abstract

Mesenchymal stromal cells (MSC) are promising candidates for regenerative therapy of the infarcted heart. However, poor cell retention within the transplantation site limits their potential. We hypothesized that MSC benefits could be enhanced through a dual-cell approach using jointly endothelial colony forming cells (ECFC) and MSC. To assess this, we comparatively evaluated the effects of the therapy with MSC and ECFC versus MSC-only in a mouse model of myocardial infarction. Heart function was assessed by echocardiography, and the molecular crosstalk between MSC and ECFC was evaluated in vitro through direct or indirect co-culture systems. We found that dual-cell therapy improved cardiac function in terms of ejection fraction and stroke volume. In vitro experiments showed that ECFC augmented MSC effector properties by increasing Connexin 43 and Integrin alpha-5 and the secretion of healing-associated molecules. Moreover, MSC prompted the organization of ECFC into vascular networks. This indicated a reciprocal modulation in the functionality of MSC and ECFC. In conclusion, the crosstalk between MSC and ECFC augments the therapeutic properties of MSC and enhances the angiogenic properties of ECFC. Our data consolidate the dual-cell therapy as a step forward for the development of effective treatments for patients affected by myocardial infarction.

## 1. Introduction

Cardiovascular disease is the leading cause of death worldwide, with myocardial infarction-based injury as one of its main hallmarks [1]. In recent times, the technological improvements in angioplasty and the treatment with new-generation drugs have reduced the mortality associated with acute myocardial infarction (MI). However, due to the massive loss of cardiomyocytes and adverse ventricular remodeling, the pumping ability of the infarcted heart diminishes progressively and eventually leads to heart failure [2].

Numerous cell-based approaches have been developed during the past 20 years aiming at improving the function of the failing heart [3,4,5]. Among them, adult mesenchymal stromal cells (MSC) have been extensively used in both preclinical and clinical investigations. Initial studies performed on animal models have shown several benefits of MSC administration on infarcted hearts in terms of scar reduction and improvement of cardiac output [6,7]. However, these benefits were only partially validated in clinical trials, thus indicating that cell-based therapy, although safe, was not very effective in the treatment of myocardial diseases [8]. 

A significant obstacle to the functional repair of the infarcted myocardium and the progress of cell-based cardiac therapies toward clinics is the poor engraftment of the transplanted cells at the site of injury [4,9]. Overall, the current opinion is that the benefits produced by transplantation of MSC to diseased hearts in various animal models occur mostly through the transient paracrine stimulation of endogenous repair mechanisms, rather than stem cell differentiation into cardiomyocytes [10]. Therefore, strategies to improve the local retention of transplanted cells into the infarcted myocardium are expected to increase the effectiveness of stem cell therapy. 

We hypothesized that a dual-cell therapy, combining two cell populations, i.e., MSC and endothelial colony forming cells (ECFC), would improve the benefits produced by transplantation of MSC-only in a mouse model of myocardial infarction. The rationale behind the dual-cell approach relies on previous results showing an enhanced ECFC engraftment and improved vascularization and blood flow when these cells were co-transplanted with MSC in mice [11,12]. Additionally, our previous studies showed that a mixture of MSC and early-outgrowth endothelial progenitor cells sustained both the adhesion and proliferation of endothelial cells in culture to an extent that neither cell type alone could support. The data suggest different yet complementary angiogenic properties of the two cell populations [13]. Considering that both the myocardial tissue and the vasculature are extensively injured after an infarct, a dual-cell-based therapy that simultaneously targets these two damaged tissues is expected to better sustain the cardiac repair process. It is therefore conceivable that, compared to MSC alone, the combinatorial use of MSC and ECFC could produce functional improvements within the failing heart. 

We present data here showing that the co-transplantation of MSC and ECFC into the myocardial wall significantly increased the function of the infarcted heart in mice. Moreover, through the analysis of the paracrine properties of these two cell types (in vitro, individually and in co-culture), we demonstrate that their cross-talk resulted in reciprocal stimulation. Therefore, we propose the dual-cell therapy as a step forward toward the development of an effective strategy for cardiac repair.

## 2. Results

### 2.1. Structural and Functional Characterization of Human MSC

Characterization of human MSC was performed according to the recommendations of The International Society for Cellular Therapy [14]. Flow-cytometry analysis showed that cultured MSC at passage six were devoid of hematopoietic cells (no staining for CD45, CD14, and CD11b), and all cells were positive for CD105, CD90, and CD73 (Figure S1A). The multi-lineage differentiation potential of the cells was confirmed by their ability to generate osteoblasts, adipocytes, and chondrocytes when cultured under appropriate experimental conditions. Differentiated cells were confirmed by specific histological stainings: von Kossa staining, which revealed the calcium deposits in osteoblasts, OilRed O staining, which indicated lipid droplets in adipocytes, and Alcian blue staining, which indicated acid mucopolysaccharides in chondrocytes (Figure S1B). 

### 2.2. Dual Transplant of MSC and ECFC Improves the Cardiac Function after Myocardial Infarction

**Figure 1:**
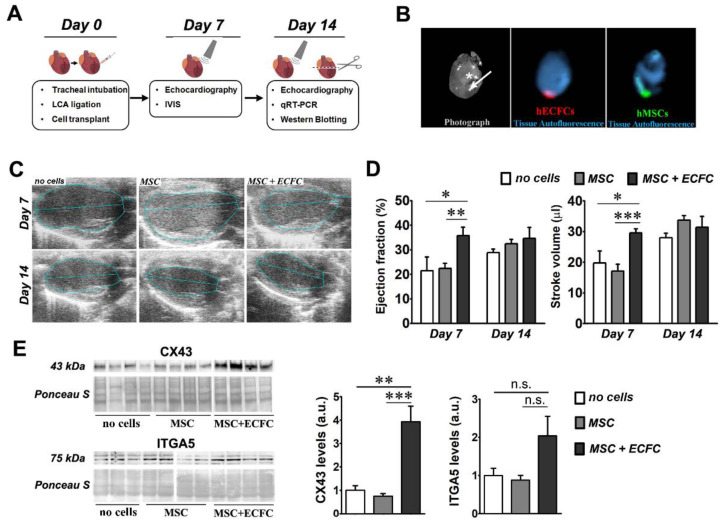
The effect of dual-cell therapy on infarcted myocardium. (**A**) Schematic representation of the experimental timeline; (**B**) ex vivo fluorescent imaging of a murine heart at 7 days after transplantation with CMFDA-labeled MSC and CMTPX -labeled ECFC at a cell ratio of 9:1 (MSC: ECFC). The white star indicates the ligature and the arrow shows the injection site and the direction of the infusion (towards the apex). Note the local retention of both MSC (green) and ECFC (red) at this time-point. (**C**) Representative echocardiography images obtained at 7 and 14 days after surgery in B-mode parasternal long axis; (**D**) ejection fraction and stroke volume determined at days 7 and 14 post-transplant. Data are mean ± SEM; (**E**) Western blot images showing the levels of CX43 and ITGA5 in the ventricular samples of infarcted mice with or without cell therapy at 14 days after surgery. The densitometry quantification relative to the no cells group is illustrated on the right side for both proteins. (Statistics: two-way and one-way ANOVA, followed by Tukey’s multiple comparison test; * *p* value < 0.05; ** *p* value < 0.01, ***p < 0.001).

We first evaluated comparatively the functional benefits of MSC therapy versus the dual-cell stem therapy, namely a combination of MSC and ECFC on infarcted hearts. As mentioned above, we designed two experimental groups in which mice with MI, induced by permanent ligation of the left coronary artery (LCA), received intra-myocardial injections with MSC (MSC group) or a mixture of MSC and ECFC (MSC + ECFC group) at a cell ratio of 9:1 (MSC: ECFC). A third group of infarcted mice was injected with phosphate-buffered saline (PBS) (no cells group) and served as the control. Cells were injected in the area below the LCA ligation site. Cardiac function was assessed by echocardiography at 7 and 14 days after ligation, and gene and protein expressions in the left ventricle were analyzed by quantitative Reverse Transcription Polymerase Chain Reaction (qRT-PCR) and Western blot (Figure 1A). 

In preliminary experiments, we monitored the post-transplant fate of the cells by ex vivo imaging analysis of the hearts. The results confirmed the presence of the fluorescently labeled cells at the transplantation site at one week after myocardial infarction (Figure 1B). No fluorescent signal, however, was noted after two weeks (data not shown).

Echocardiography analysis at 7 days after cell injection revealed that MSC-only therapy induced no significant improvement over the no cells group, in accordance with results presented elsewhere [15,16]. However, a significant increase was observed in the ejection fraction (EF) of the hearts in the MSC + ECFC group (35.7 ± 3.5%) as compared to MSC (22.4 ± 2.1%) and no cells (21.5 ± 5.6%) (Figure 1C,D). This improvement was only transient, as the difference in the ejection fraction between the three groups was non-significant at 14 days after infarction. Similar transient effects were observed in the stroke volume (SV) at 7 days post-transplantation, with 29.6 ± 1.3 µL for the MSC + ECFC group, as compared to 17.1 ± 2.2 µL in the MSC group and 19.7 ± 3.9 µL in the no cells group. Additional parameters were also determined (end-systolic volume (ESV), end-diastolic volume (EDV), and fractional shortening (FS)), but no significant changes were detected among the three experimental groups (Figure S2). These data suggested the dual-cell therapy promoted a quicker functional recovery rather than a robust regeneration of the infarcted heart.

### 2.3. Dual-Cell Therapy Results in Increased Expression Levels of Connexin 43 and Integrin Alpha-5 in the Infarcted Heart

As Connexin 43 (CX43) and Integrin alpha-5 (ITGA5) fibronectin receptor subunits play important roles in connecting cardiac cells to each other and to the extra-cellular matrix (ECM) [17,18], we next evaluated whether the dual-cell therapy affected their expression in the infarcted hearts. Western blot analysis (Figure 1E) indicated an increased CX43 protein level in the MSC + ECFC group (3.93± 0.66-fold increase over no cells group), yet not in the MSC group (0.75 ± 0.11-fold change versus no cells group). In a similar trend, increased protein levels of ITGA5 were detected in the MSC + ECFC group as compared to the MSC and no cells groups (2.3 ± 0.57-fold increase in MSC + ECFC group and 0.97 ± 0.17-fold change in MSC group over no cells group). However, the ITGA5 increase did not reach statistical significance. Similar trends were also found in the mRNA level for both genes (Figure S3). These data suggested that ECFC co-transplanted with MSC enhanced local cell-to-cell communication and cellular integration within the extracellular matrix.

Based on our previous studies showing improved CX43-mediated intercellular communication between cardiomyocytes and MSC after MSC treatment with fibroblast growth factor 2 (FGF-2) [19], we next questioned whether the local FGF-2 expression was stimulated by the dual-cell therapy. The qRT-PCR data indicated an increased expression of FGF-2 mRNA levels in the MSC + ECFC group over the MSC group (Figure S3). Specifically, a 4.23 ± 0.56-fold increase in the expression level of *FGF-2* over the no cells group was noted in the MSC + ECFC group versus 2.99 ± 0.25 in the MSC group. Therefore, we acknowledge that the local increase in *FGF-2* expression might contribute to the improved outcomes of the dual-cell therapy, as FGF-2 reportedly exerts pro-survival and cardioprotective roles and stimulates inter-cellular communication between MSC and cardiomyocytes via CX43 [20,21,22].

### 2.4. MSC and ECFC Secrete Distinct Factors with Angiogenic Properties

**Figure 2:**
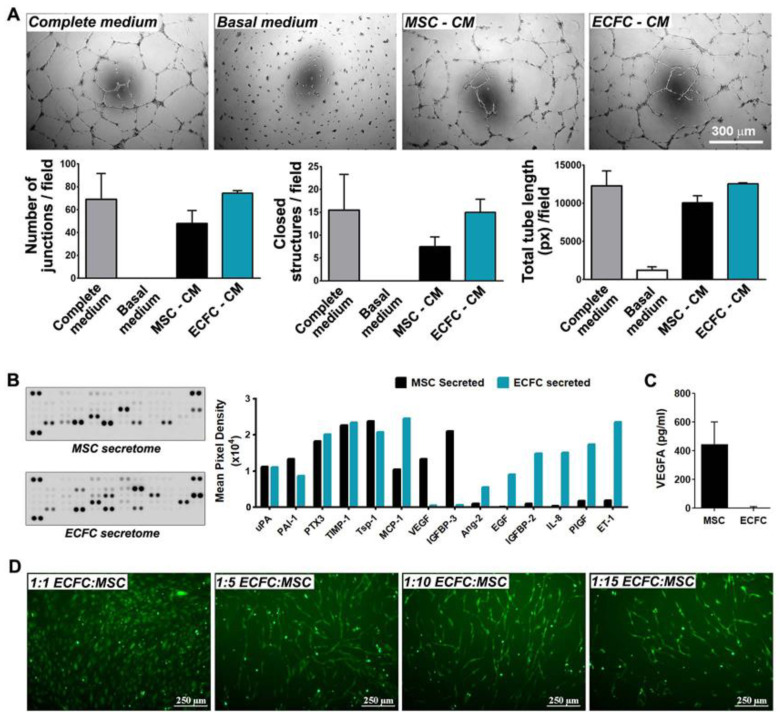
Angiogenic effects of MSC and ECFC. (**A**) Phase-contrast microscopy images showing vascular networks of endothelial cells assembled on Matrigel in the presence of MSC- and ECFC-CM. The diagrams illustrate comparable effects of the MSC- and ECFC-CM in terms of the number of junctions, total tube length, and closed structures formed by endothelial cells in one representative experiment performed in duplicates; (**B**) proteomic screening of cytokines secreted by MSC and ECFC in vitro, as assessed by Human Angiogenesis Proteome Profiler array; (**C**) ELISA quantification of VEGF in MSC and ECFC secretomes; (**D**) tube-like structures formed by fluorescent ECFC when co-cultured with unlabeled MSC in different cell ratios.

We previously showed that MSC and ECFC played important complementary roles and improved the outcome of angiogenic therapy in ischemic tissues [13,23]. To assess the angiogenic potential of human MSC and ECFC, the capacity of their secretomes to induce the organization of endothelial cells in tube-like structures was evaluated by in vitro Matrigel assay. To this aim, endothelial cells were seeded onto the Matrigel layer in the presence of MSC-conditioned medium (MSC-CM) or ECFC-conditioned medium (ECFC-CM), and the assembled structures were quantified 24 h later. The results indicated that the secretome of both cell types, harvested in the absence of fetal bovine serum (FBS), supported the assembly of endothelial cells in vascular networks at levels comparable to complete growth medium containing FBS (Figure 2A). As expected, no network formed in the basal medium. The number of junctions formed in the presence of MSC-CM (48 ± 11.3 junctions/field) or ECFC-CM (74.5 ± 2.1 junctions/field) closely approximated the values observed in the complete medium (69 ± 22.6 junctions/field). Similar results were obtained in terms of total length of vascular networks (10,076.0 ± 912.2 pixels/field for MSC-CM and 12,540.5 ± 120.9 pixels/field for ECFC-CM, as compared to 12,283.5 ± 1959.4 pixels/field for complete culture medium) and closed structures (15 ± 2.8 structures/field for ECFC-CM and 7.5 ± 2.1 structures/field for MSC-CM). These data indicated that MSC and ECFC have important proangiogenic properties in vitro.

The cytokines released by MSC and ECFC in their secretomes were next determined by a human angiogenesis proteome profiler array. The screening against a set of 55 angiogenic molecules revealed 14 proteins to be notably abundant in the secretome of at least one cell type (Figure 2B). Six of these proteins (uPA, PAI-1, PTX3, TIMP-1, Tsp-1, and MCP-1) were found to be secreted by both MSC and ECFC, being previously reported to contribute to several important biological processes such as inflammatory response, extracellular matrix remodeling, and neovessel formation [24,25,26,27,28]. Specifically, the secretion of urokinase-type plasminogen activator (uPA) and its inhibitor, Plasminogen Activator Inhibitor-1 (PAI-1), both part of the plasminogen activator proteolytic enzyme system, suggested the ability of the cells to regulate ECM degradation, a crucial process for the initiation of angiogenesis [24]. Additionally, uPA has been shown to induce the release of different types of proangiogenic growth factors, such as Vascular Endothelial Growth Factor (VEGF) and FGF-2, which play key roles in endothelial cell proliferation and invasion [24,25]. High levels of secreted metallopeptidase inhibitor-1 (TIMP-1) also pointed to the paracrine ability to regulate cell invasion, considering its roles in metalloproteinase inhibition and matrix accumulation [26]. Moreover, the secretome profile showed a balanced secretion of pro- and anti-inflammatory molecules, such as Monocyte Chemoattractant Protein-1 (MCP-1) and Thrombospondin-1 (Tsp-1), respectively. Tsp-1 shares an anti-angiogenic role with Pentraxin-3 (PTX3) [27], the latter being shown to have inhibitory effects on FGF-2 and to prevent angiogenesis through multiple mechanisms [28]. 

Besides the molecules secreted by both cell types, the array distinguished two proteins, namely VEGF and Insulin-like growth factor-binding protein 3 (IGFBP-3), which were secreted by MSC and not by ECFC, indicating an additional pro-angiogenic potential of MSC over ECFC [29,30]. The absence of VEGF in the ECFC secretome was validated by an enzyme-linked immunosorbent assay (ELISA) (Figure 2C). With regards to molecules secreted exclusively by ECFC, these could also largely be divided into pro-angiogenic, i.e., epidermal growth factor (EGF), Insulin-like growth factor-binding protein 2 (IGFBP-2), placental growth factor (PIGF), and Endothelin-1 (ET-1) [31,32,33,34]; anti-angiogenic, i.e., Angiopoietin-2 (Ang-2) [35]; and pro-inflammatory cytokines, i.e., Interleukin-8 (IL-8). Together, these data indicated different secretory profiles of the two cell types, with a partial overlap in the angiogenic proteins that could explain the enhanced effects obtained with dual-cell therapy, in comparison to MSC mono-therapy.

As an assay to estimate the stimulatory effect of MSC on the angiogenic properties of ECFC, we evaluated the ECFC alignment when co-cultured in direct contact with MSC. For this, 5-chloromethylfluorescein diacetate (CMFDA)-labeled ECFC were co-cultured with MSC and analyzed by fluorescence microscopy 6 days later. The results showed that mixing the two cell types at 1:5 and 1:10 ECFC:MSC ratios resulted in the positioning of ECFC as cellular networks (Figure 2D). In contrast, a low number of ECFC (i.e., 1:15 ratio) led to a reduced capacity to form similar structures in vitro, while higher numbers of ECFC (i.e., 1:1 ratio) resulted in no alignment pattern. The capacity of ECFC to self-assemble in tube-like structures in vitro alongside MSC may be indicative of their proclivity to organize into blood vessels when transplanted together in vivo.

### 2.5. Interaction with ECFC Induces the Upregulation of Fibronectin Receptor in MSC

**Figure 3:**
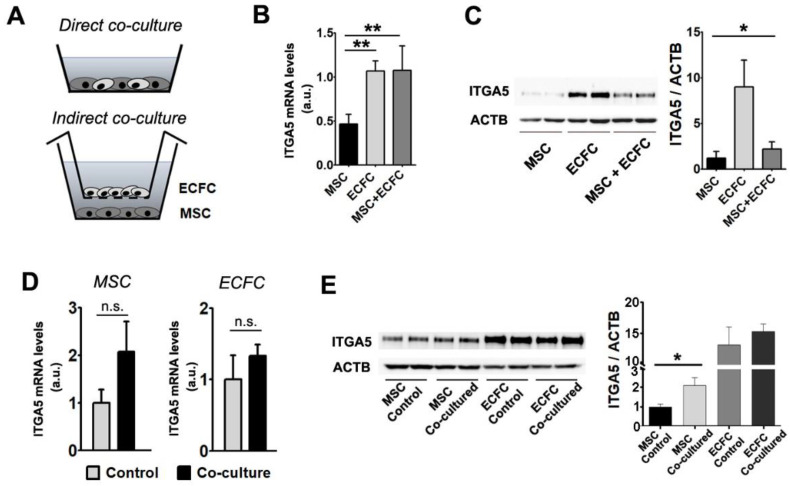
Gene and protein expression modulation following MSC + ECFC co-culture. (**A**) Schematics of the experimental settings used for MSC + ECFC direct contact (upper image) and indirect co-culture (lower image). (**B**) Gene expression analysis of *ITGA5* in individual cultures and MSC + ECFC direct co-culture, indicating the induction after co-culture. (**C**) Western blot showing protein levels of Integrin alpha-5 subunit after direct MSC–ECFC interaction. (**D**) Gene expression analysis of *ITGA5* in MSC (left side) and ECFC (right side), in individual cultures (control) and after indirect-contact co-culture. (**E**) Western blot showing ITGA5 protein level in MSC and ECFC following indirect contact co-culture. Data are mean ± SD. (Statistics: one-way ANOVA followed by Tukey’s multiple comparison test and two-tailed, paired *t* test; * *p* value < 0.05; ** *p* value < 0.01).

The reciprocal modulation of MSC and ECFC was studied in direct and indirect co-cultures (Figure 3A). For direct co-cultures, cell suspensions containing either mixtures of MSC and ECFC (at 1:1 ratio) or monocellular controls were seeded at confluency. After 24 h, the pooled cells (a mixture of MSC and ECFC) and the mono-culture controls were comparatively analyzed for ITGA5 at both mRNA and protein expression levels (Figure 3B,C). The gene expression analysis indicated that ITGA5 mRNA levels were 2 times higher in ECFC than in MSC (Figure 3B). Moreover, *ITGA5* gene expression in the co-cultured cells was higher than expected, considering the expression in the mono-culture controls (1.07 ± 0.27-fold change for MSC + ECFC versus 0.46 ± 0.1-fold change for MSC and 1.06 ± 0.11 for ECFC) (Figure 3B). Western blot analysis indicated trace levels of ITGA5 in MSC, yet an abundance of this protein in ECFC. These data warrant the advantage brought by ECFC in coculture settings in order to reach a sustained level of ITGA5. Indeed, the pooled cells exhibited a slight increase in ITGA5 level as compared to MSC mono-culture (1.8 ± 0.6-fold increase in MSC + ECFC over MSC) (Figure 3C). Although the total ITGA5 protein level in co-cultured cells did not reflect the expected value considering the high ECFC expression, the presence of ECFC grants an additional benefit within the co-culture.

We next questioned whether the direct contact between the two cell types was necessary for the observed effects or whether the same benefits could be obtained by indirect contact. To this aim, the two cell types were co-cultured in a Transwell system, which allowed the exchange of secreted factors but not the contact between ECFC and MSC. After 24 h of co-incubation, mRNA and protein levels of ITGA5 were assessed in each cell type (Figure 3D,E). The results indicated that MSC, but not ECFC, maintained in co-culture, expressed a 2.07 ± 0.64-fold increase in *ITGA5* gene expression level, as compared to the corresponding mono-culture control (Figure 3D). This variation, although not statistically significant, resulted in a 2.1 ± 0.5-fold increase in protein level of ITGA5 in MSC after co-culture as compared to the MSC control (Figure 3E). 

These results suggested that ECFC modulate MSC properties through multiple pathways, both dependent and independent of cell-to-cell contact. Considering that ITGA5 is directly involved in cell adhesion to fibronectin and mediates the linkage to the cytoskeletal structures, our results project an improved ability of the co-transplanted cells to engraft into the host tissue [17,36].

### 2.6. The Direct Contact between MSC and ECFC Stimulates the Secretion of Cytokines

**Figure 4:**
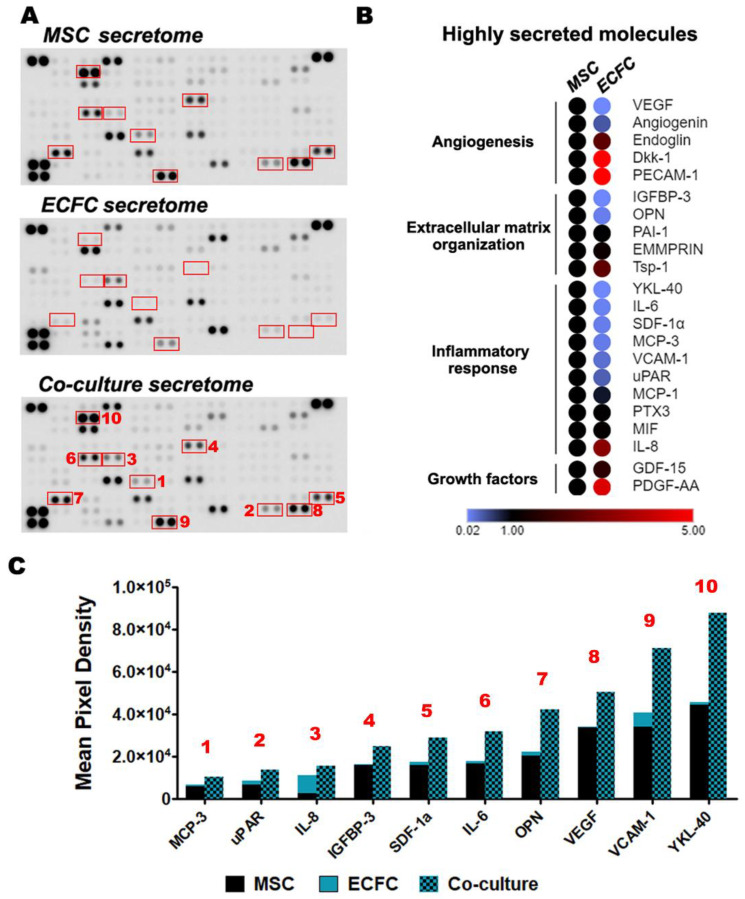
Proteome array analysis of the secretome produced by MSC and ECFC co-cultured in direct contact. (**A**) Secretory profiles of MSC, ECFC, and MSC + ECFC co-cultures were identified by antibody-based Human XL Cytokine Array. Shown in red are proteins whose cumulative levels were induced following co-culture as compared to their expected levels; (**B**) heatmap representation of the highly secreted cytokines, illustrating the major secretion source and biological processes associated to the cytokines; (**C**) the relative levels of proteins in the co-culture supernatant, which were found to be induced by more than 35% after MSC-ECFC co-culture.

Considering that in vivo results made use of MSC and ECFC in close contact and that in co-culture experiments, direct cell to cell contact showed improved cell properties over indirect contact, we further interrogated the factors that contribute to the beneficial effects obtained in dual-cell therapy. To this aim, MSC and ECFC were co-cultured in direct contact at 1:1 ratio, and the resulting secretome was analyzed by a Human XL Cytokine Array Kit against a set of 105 soluble human proteins. To minimize the influence of cell density, an identical number of cells was used in all experimental conditions, meaning that the number of each cell type in the co-culture group was half the number of cells in the respective mono-culture control. The signal analysis was done by normalizing to cell number, and a molecule was considered to be induced when the signal in the co-culture sample exceeded the sum of the contribution of each cell type by at least 35%. The results enabled the distinction between highly secreted proteins as well as many cytokines with lower abundance (Figure 4A). 

Upon analysis of the most abundant cytokines, the angiogenesis, extracellular matrix organization, and inflammatory response were found to be the overrepresented pathways. These results suggested the ability of MSC and ECFC to modulate wound-healing processes through their secreted factors (Figure 4B). Several of these molecules were highly secreted by both MSC and ECFC in similar amounts: Angiogenin, Dickkopf-related protein 1 (Dkk-1), Extracellular Matrix Metalloproteinase Inducer (EMMPRIN), Endoglin, Growth/Differentiation Factor 15 (GDF-15), MCP-1, Macrophage Migration Inhibitory Factor (MIF), PTX3, PAI-1, Thrombospondin-1, and the Platelet And Endothelial Cell Adhesion Molecule 1 (PECAM-1) (Figure S4). In contrast, two molecules, Dkk-1 and PECAM-1, with previously reported proangiogenic activity, were contributed only by ECFC. 

Although these data may not have sufficient power in the absence of ELISA validation for each individual molecule, it is interesting to note that ten secreted molecules [Monocyte-Chemotactic Protein 3 (MCP-3), urokinase Plasminogen Activator Surface Receptor (uPAR), IL-8, IGFBP-3, Stromal Cell-Derived Factor 1 (SDF-1a), Interleukin 6 (IL-6), Osteopontin (OPN), VEGF, Vascular Cell Adhesion Molecule 1 (VCAM-1), and Chitinase-3-like Protein 1 (YKL-40)] appeared to be induced in the co-cultured cells, as compared to the monocultured controls, by at least 35% (Figure 4A, red rectangles). The induction was more obvious for OPN and YKL-40, which exceeded the expected secretion by 88% and 92%, respectively (Figure 4C). Of note, OPN has a protective in vivo role by maintaining the heart function post-MI and has been shown to positively influence local remodeling processes, whereas YKL-40 was shown to contribute to wound healing and fibrosis [37,38]. Although ECFC did not directly contribute substantial amounts of either OPN or YKL-40 in monoculture, their boosted secretion levels following the co-culture suggested a reciprocal modulation between MSC and ECFC. Similarly, MSC + ECFC co-culture samples indicated stimulated VEGF secretion compared to either monocultured control. Given the importance of neovascularization processes within the infarcted myocardium, stimulation of local VEGF secretion by transplanted cells could enhance the dual-cell therapy’s contribution to tissue repair [39]. With the exception of IL-8, all of the above-mentioned induced molecules appear to have been preferentially contributed by MSC, thus supporting the hypothesis of a reciprocal, stimulating effect between MSC and ECFC.

## 3. Discussion

Numerous efforts have been undertaken to stimulate heart regeneration following myocardial infarction. While the overall safety of cell therapies has been repeatedly confirmed [40], their clinical efficacy is still controversial and the underlying mechanisms are incompletely understood [4]. This study reveals that the outcome of MSC-based therapy for myocardial infarction can be improved through a combined approach using jointly ECFC and MSC. 

A major drawback of cell-based therapies is the poor retention of the transplanted cells within the host tissue, likely due to the hostile micro-environment for engraftment, which limits the time window of the beneficial effects [5]. Despite these limitations, several preclinical studies indicated an overall advantage of intra-myocardial transplantation of stem cells compared to alternative routes such as intra-coronary and intra-venous administration [41]. 

The results of our experiments show that the crosstalk between MSC and ECFC augments the effector properties of MSC. Indeed, their concomitant use resulted in an increase in the ejection fraction and stroke volume in infarcted mice, indicating improved cardiac function. The stimulatory effect of ECFC on MSC was revealed by the increased expression of Connexin 43 and Integrin alpha-5 in animals receiving the dual-cell therapy compared to those receiving MSC only. These proteins mediate intercellular and cell-extracellular matrix couplings, with important roles in integrating the exogenously delivered cells within the host tissue [17,18]. It is, however, uncertain whether the observed effect occurred in cardiomyocytes or within fibroblast populations. Nevertheless, our in vitro results confirm that the molecular crosstalk between MSC and ECFC leads to increased Integrin alpha-5 expression in MSC, in a fashion independent of cell-to-cell contact, therefore projecting an improved ability of the co-transplanted cells to engraft at the transplantation site. Future histological analysis could provide insights into the structural changes associated with the improved outcome observed in the dual-cell therapy. The MSC and ECFC mixture has previously been explored in co-transplantation studies and the results indicated that the presence of ECFC significantly enhanced MSC engraftment by reducing early apoptosis [42]. Similar results were reported by Shafiee et al., indicating a reciprocal stimulation between MSC and ECFC, with MSC co-implantation improving the ECFC engraftment by stimulation of the vasculogenic and pro-angiogenic activities in immunocompetent mice [11]. 

In our study, we identified that each cell type, MSC and ECFC, secreted factors with pro-angiogenic properties and the respective secretomes only partially overlapped. Therefore, it is safe to assume that a dual approach using this particular combination of cell types could promote an improved cardiac repair in the setting of myocardial infarction. The cross-talk between MSC and ECFC and the ensuing modulated secretory activity observed in this study could possibly stimulate in vivo effects such as local angiogenesis (likely through VEGF, IGFBP-3, and YKL-40) [29,43], protection of cardiomyocytes against apoptosis (likely through IGFBP-3) [44], or prevention of left ventricular dilation post-MI (likely through OPN) [37]. 

In addition, our data show that not only do ECFC stimulate MSC properties, but MSC also assist ECFC to organize into vascular networks. All these results suggest a reciprocal modulation of the functionality of these two cell types following their interaction. MSC, through their secretory activity, have been reported to stimulate new blood vessel formation, activate endogenous cell repair programs, and induce adhesion and chemotaxis of endothelial cells in vitro [13,45,46]. Based on our previous studies confirming the synergistic protective and pro-angiogenic effects of endothelial progenitor cells (EPC) in conjunction with MSC, we assume that ECFC could assist cardiac regeneration by paracrine activation of the local angiogenesis, while MSC could sustain the survival of partially damaged cardiac cells [13,47,48,49]. Our results are in good agreement and extend the existing body of literature reporting intense paracrine activity of MSC [2,10,50] as well as the ability of ECFC to form vascular structures in vitro [51]. 

In conclusion, the dual-cell therapy for myocardial infarction based on joint administration of MSC and ECFC supports a faster recovery, most likely through a better engraftment and the paracrine contribution of both cell types. This study consolidates the MSC + ECFC therapy in particular, and a dual-cell therapy in general, as a step forward in the development of more effective cell-based treatments for myocardial infarction. Future work is expected to provide a wider understanding of the modulations occurring within MSC and ECFC following their interaction by transcriptome analysis studies such as RNA-Sequencing. Such transcriptomic data will add to other already reported complex network analyses of MSC in dialogue with other cells to better predict the outcomes of MSC therapy and accelerate the translation toward precision medicine [52,53].

## 4. Materials and Methods

### 4.1. Animal Studies

All animal experiments were conducted in accordance with the European Guidelines for Animal Welfare (Directive 2010/63/EU) and approved by the National Sanitary Veterinary and Food Safety Authority (nr 330/10.07.2018). Mice were maintained under specific pathogen-free conditions in a controlled environment with a 12/12-h light/dark cycle, 21 °C, and 55–60% humidity, and had access to chow and water ad libitum.

Myocardial infarction was induced by left coronary artery ligation, as previously described [54]. Briefly, three-month-old male and female NOD scid gamma (NSG) mice (Jackson Laboratory, Bar Harbor, ME, USA) were anesthetized with ketamine/xylazine (100/20 mg/kg body weight) and intubated orotracheally by cannulating the trachea with a 20-gauge blunt needle attached to a mouse ventilator via a Y-connector. Hearts were exposed by left thoracotomy, and MI was induced by permanent suture occlusion of the LCA. The ligation was confirmed by the paleness of the apex. Immediately after LCA ligation, 5 µL of cell suspension was injected into the ventricular wall, just below the ligature site using a 33-gauge needle. Sham-operated mice were used as controls. The chest, muscles, and skin were then closed with 6-0 polypropylene threads, and mice were allowed to recover on a heating plate at 37 °C. For analgesia, all animals received subcutaneous injections of buprenorphine hydrochloride (Temgesic^®^, 0.1 mg/kg body weight) prior to surgery and on the first post-operative day. Mice were randomly assigned to 3 experimental groups: (a) MI + no cells (control mice that underwent LCA ligation and only basal medium injection; *n* = 4), (b) MI + MSC (mice that underwent LCA ligation and MSC injection; *n* = 7), and (c) MI + MSC + ECFC (mice that underwent LCA ligation and MSC + ECFC injection; *n* = 7). Two weeks after surgery, animals were anesthetized using ketamine/xylazine. The hearts were excised, washed in cold phosphate buffer saline, cut above the ligature and the apex, and then snap-frozen in liquid nitrogen. Homogenized hearts were resuspended in radioimmunoprecipitation assay buffer (RIPA buffer) for downstream Western blot analysis or lysis buffer for total RNA isolation.

### 4.2. Echocardiography Analysis

Was performed using a Vevo2100 ultrasound system (Visualsonics, Toronto, ON, Canada) equipped with a 30 MHz MicroScan transducer. Mice underwent isoflurane anesthesia, and the measurements were obtained in B-mode images of the parasternal long- and short-axis. Echocardiographic analysis was performed at 7 and 14 days after LCA ligation surgery. The left ventricular ejection fraction, stroke volume, end-systolic volume, end-diastolic volume, and fractional shortening were determined. Animals with cardiac parameters indicative of healthy, non-infarcted hearts that also presented no histological signs of MI upon visual examination were excluded from the study.

### 4.3. In Vivo Tracking of Transplanted Cells

Cell engraftment was evaluated by in vivo imaging of fluorescently labeled cells at 7 and 14 days post-transplantation, using the IVIS Spectrum In Vivo Imaging System (PerkinElmer, Waltham, MA, USA). Briefly, MSC and ECFC were incubated with 10 µM CellTracker Green CMFDA Dye (Life Technologies, Waltham, MA, USA) and CellTracker Red CMTPX Dye (Invitrogen, Waltham, MA, USA), respectively, according to the manufacturer’s instructions. Subsequently, the cells were washed and injected into the myocardial wall immediately after LCA ligation, as described above. One week after cell transplant, the heart was excised, washed in PBS, and evaluated by IVIS. Images were analyzed with Living Image 4.5 software (PerkinElmer, Waltham, MA, USA) using spectral unmixing analysis to separate the signals of the two fluorophores and extract the auto-fluorescence. Fluorescence signal calculated as total radiant efficiency was illustrated in pseudocolors green (for CMFDA dye), red (for CMTPX dye), and blue (for tissue autofluorescence). 

### 4.4. Cell Culture

Human endothelial colony forming cells were generated from umbilical cord blood collected at the time of delivery as previously described [23]. Cells were routinely cultured in endothelial cell growth medium (EGM-2) complete culture medium (Lonza, Switzerland) on rat tail collagen type 1-treated plates. The cells were used between the 6th and 11th passages.

Human mesenchymal stromal cells were obtained from bone marrow aspirates of patients undergoing clinical investigations upon written informed consent for the collection, analysis, storage, and reuse. Mononuclear cells were isolated using a Ficoll density gradient and cultivated on gelatin-treated cell culture dishes, in 1 g/L glucose Dulbecco’s Modified Eagle Medium (DMEM) supplemented with 10% MSC-qualified fetal bovine serum (Gibco, Waltham, MA, USA). Cells were used between the 6th and 11th passages. For in vivo studies, the cells were trypsinized and re-seeded at confluence for 24 h before use in the cellular transplant. On the transplantation day, the cells were detached with accutase (Sigma-Aldrich, St. Louis, MO, USA), and suspensions were prepared containing 40 × 10^6^ cells/mL. Five microliters of cell suspension, containing 2 × 10^5^ cells (for the MSC group), or a mixture of 1.8 × 10^5^ MSC and 2 × 10^4^ ECFC (for the MSC + ECFC group) was used per mouse.

### 4.5. Assessment of the Tri-Lineage Differentiation Potential of MSC

The cells were cultured on 6-well plates until confluence, then incubated in the differentiation medium for two weeks. Adipogenesis was induced in low glucose DMEM supplemented with 10% MSC-qualified FBS, 10^−6^ M dexamethasone, 100 µM indomethacin, and 1% ITS (insulin-transferrin-sodium selenite supplement). Osteogenesis was induced in low glucose DMEM supplemented with 10% MSC qualified-FBS, 10^−7^ M dexamethasone, 10 mM β-glycerophosphate, and 0.3 mM ascorbic acid. Lipid droplets were highlighted by Oil Red staining, and calcium deposits by von Kossa staining.

Chondrogenic differentiation was induced in high-glucose DMEM supplemented with 1% ITS, 100 nM dexamethasone, 100 µM ascorbic acid-2-phosphate, and 10 ng/mL TGFβ3. Briefly, 2 × 10^5^ cells were resuspended in 500 µL differentiation medium in a 15-mL tube, centrifuged at 400× *g* for 5 min, then cultured as pellets for three weeks at 37 °C. Culture medium was changed every 3 days. The pellets were eventually embedded in paraffin, and the 5-µm-thin sections were stained with Alcian Blue (1%).

### 4.6. Flow Cytometry Analysis

Trypsinized cells were resuspended at 10^6^ cells/mL in 2% FBS in PBS. For each analysis, 100 µL cell suspension was incubated with an appropriate dilution of specific or isotype-matched control fluorescent antibody for 30 min on ice, then washed and centrifuged at 400× *g* for 5 min. Cells were then resuspended in 250 µL 2% FBS/PBS in the presence of 2 µg/mL propidium iodide and analyzed using a CytoFlex cytometer (Beckman Coulter, Indianapolis, IN, USA). At least 20,000 events were recorded for each sample. Acquired data were analyzed using CytExpert software (Indianapolis, IN, USA).

### 4.7. In Vitro Assessment of MSC and ECFC Direct and Indirect Dialogue (Co-Culture Experiments)

MSC and ECFC were co-cultured as a mixed culture (with direct contact) or in a dual-chamber system (with indirect contact). For direct contact co-cultures, a total of 6 × 10^4^ cells/cm^2^ were seeded at 1:1 cell ratio on collagen-treated plates in basal low glucose DMEM supplemented with 0.5% bovine serum albumin. Monocellular cultures were used as controls. After 24 h of direct contact in co-culture, the supernatant (secretome) was collected and analyzed, and the cells were lysed in appropriate lysis buffer according to the downstream analysis.

In the case of indirect co-culture, MSC were seeded at 5500 cells/cm^2^ on the bottom plate and ECFC (5500 cells/cm^2^) were plated on the Transwell of a dual-chamber system (pore size of 0.4 µm) and cultured in separate wells until they reach confluence (96 h). Then, the transwell system was assembled, and the cells were co-cultured for 24 h in basal, low-glucose DMEM. The monocellular cultures used as controls consisted of the assembled Transwell system with the same cell type seeded on both chambers. 

### 4.8. Preparation of the Secretome

Conditioned medium (CM) was collected from direct contact co-cultures, as well as from MSC and ECFC mono-cultures. Prior to harvest, all cells were incubated in basal media for 24 h to allow secretome release in the absence of fetal bovine serum and its constituent proteins. The cell supernatant was then centrifuged at 400× *g* for 5 min at room temperature and subsequently at 2000× *g* for 25 min at room temperature to remove cell debris. The medium thus prepared was used for secretome analysis by ELISA, Proteome Profiler assays, and Matrigel assays. 

### 4.9. In Vitro Matrigel Assay

Liquid Matrigel matrix (BD Biosciences, San Jose, CA, USA) was added to 96-well cell culture plates (50 µL/well) and solidified by incubation at 37 °C for 1 h. A cell suspension containing 10^4^ human endothelial cells (EAhy926 human cell line) in 100 µL EGM-2 was seeded onto each well and allowed to adhere for 4 h. The culture medium was then changed to MSC-CM and ECFC-CM. The complete medium containing FBS (EGM-2) and basal medium (EBM-2) were used as positive and negative controls, respectively. After 24 h of incubation, the cultures were examined and photographed under a ZEISS Axio Vert A.1 microscope, and the closed structures, branching points, and total tube length formed were determined using ImageJ software (National Institutes of Health, NIH, Bethesda, MD, USA).

### 4.10. Effect of MSC on the Assembly of ECFC into Tube-Like Structures

ECFC were fluorescently labeled with Green CMFDA Dye (Life Technologies, Waltham, MA, USA) by incubation with 10 mM CellTracker, according to the manufacturer’s instructions. Mixed co-cultures of MSC and fluorescent ECFC were seeded at different cell ratios (1:1, 1:5, 1:10, and 1:15, ECFC:MSC). A constant number of 6 × 10^4^ MSC/cm^2^ and varying numbers of fluorescent ECFC were seeded simultaneously on collagen-coated plates, and the co-cultures were maintained in EGM-2 medium for 6 days before being imaged under a Nikon Ti-E fluorescence microscope.

### 4.11. Proteome Profiler Assays

The relative levels of secreted cytokines into the secretome were detected using a Human Angiogenesis Array and Human XL Cytokine Array Kit (R&D Systems, Minneapolis, MN, USA). Briefly, the secretome was incubated overnight with the membranes spotted with specific capture antibodies. Excess material was removed by washing, and the membranes were incubated with a cocktail of biotinylated detection antibodies, followed by Streptavidin-HRP. The chemiluminescent detection was performed using a Luminescent Image Analyzer LAS-3000 (FUJIFILM, Japan), and mean pixel densities of all spots were determined using TotalLab Quant software (United Kingdom). Proteins with a mean pixel density over 10,000 were considered to be highly secreted. Heatmap was generated using Morpheus software (Cambridge, MA, USA) (https://software.broadinstitute.org/morpheus). 

### 4.12. ELISA Assay

Vascular endothelial growth factor concentration was quantified using a human-specific DuoSet ELISA kit (R&D Systems, Minneapolis, MN, USA), according to the manufacturer’s instructions.

### 4.13. Quantitative Real-Time RT-PCR

Total RNA was purified from samples using the PureLink RNA Mini Kit (Invitrogen, Waltham, MA, USA) and quantified by reading the absorbance at 260 nm using the Nanodrop ND-1000 Spectrophotometer (Marshall Scientific, Hampton, NH, USA). Reverse transcription was performed using the SensiFAST cDNA Synthesis Kit (Bioline, Cincinnati, OH) according to the manufacturer’s instructions, starting from 0.5 µg total RNA per reaction. Real-time PCR was performed with SYBR Select Master Mix (Applied Biosystems, Foster City, CA, USA) on a 7900HT Fast Real-Time PCR System (Applied Biosystems). Primers were designed using NCBI Primer-BLAST according to the species of interest. The list of primer sequences is given in Table S1. Data analysis was performed using the comparative CT method, and the normalization was made by reference to Glyceraldehyde 3-phosphate dehydrogenase (GAPDH) for in vitro studies or 60S ribosomal protein L32 (RPL32) for in vivo studies.

### 4.14. Western Blot

Cells were lysed in ice-cold RIPA buffer containing proteases inhibitor cocktail (Sigma), and protein concentration was determined using the Amido Black assay. Equal amounts of protein were loaded and resolved on 10% SDS-PAGE gels, followed by transfer on 0.45 µm nitrocellulose membrane and Ponceau S reversible staining. Following incubation with StartingBlock PBS blocking buffer (Thermo Fisher Scientific, Waltham, MA, USA), the membranes were incubated with anti-Integrin alpha5 antibody (Thermo Fisher Scientific PA5-12507), anti-Connexin 43 (Millipore AB1727), or anti-beta Actin (Sigma A2228) over-night at 4 °C. Membranes were rinsed in Tris-buffered saline solution and incubated with HRP-conjugated F(ab’)₂ fragment secondary antibody (Jackson ImmunoResearch, West Grove, PA, USA) for 1 h. Protein bands were imaged after enhancement with chemiluminescence agent (Thermo Fisher Scientific, Waltham, MA, USA) using an ImageQuant LAS4000 system (Fujifilm, Japan). The protein expression was quantified by densitometry with ImageJ software and normalized to beta actin or total protein as quantified by Ponceau S stain.

### 4.15. Statistical Analysis

In vivo data are presented as mean ± SEM, and in vitro data are presented as mean ± SD, as indicated in the figure legends. The statistical significance of the differences between two groups was calculated using two-tailed *t*-tests. When more than two groups were analyzed, one-way or two-way ANOVA testing was performed, followed by Tukey’s multiple comparison test. Data were analyzed using GraphPad Prism software (San Diego, CA, USA) and a *p* value less than 0.05 was considered statistically significant.

## Data Availability

The data that support the findings of this study are available from the corresponding author upon reasonable request.

## Supplementary Materials

The following are available online at https://www.mdpi.com/article/10.3390/ijms22115631/s1.
