# Supplementary material for: Dual Stem Cell Therapy Improves the Myocardial Recovery Post-Infarction through Reciprocal Modulation of Cell Functions
Source: Int J Mol Sci. 2021 May 26;22(11):5631. doi: 10.3390/ijms22115631 (PMC8199446; doi:10.3390/ijms22115631)
Supplement: Supplementary file 1 [file ijms-22-05631-s001.zip › ijms-1229626 SL.pdf]

Dual Stem Cell Therapy Improves the Myocardial Recovery Post-infarction through Reciprocal Modulation of Cell Functions

Supplementary Materials

Sinziana Popescu, Mihai Bogdan Preda, Catalina Iolanda Marinescu, Maya Simionescu and Alexandrina Burlacu [1], *

1 - Institute of Cellular Biology and Pathology "Nicolae Simionescu", 8, B.P. Hasdeu Street, 050568 Bucharest, Romania; *Correspondence: sanda.burlacu@icbp.ro (A.B.)

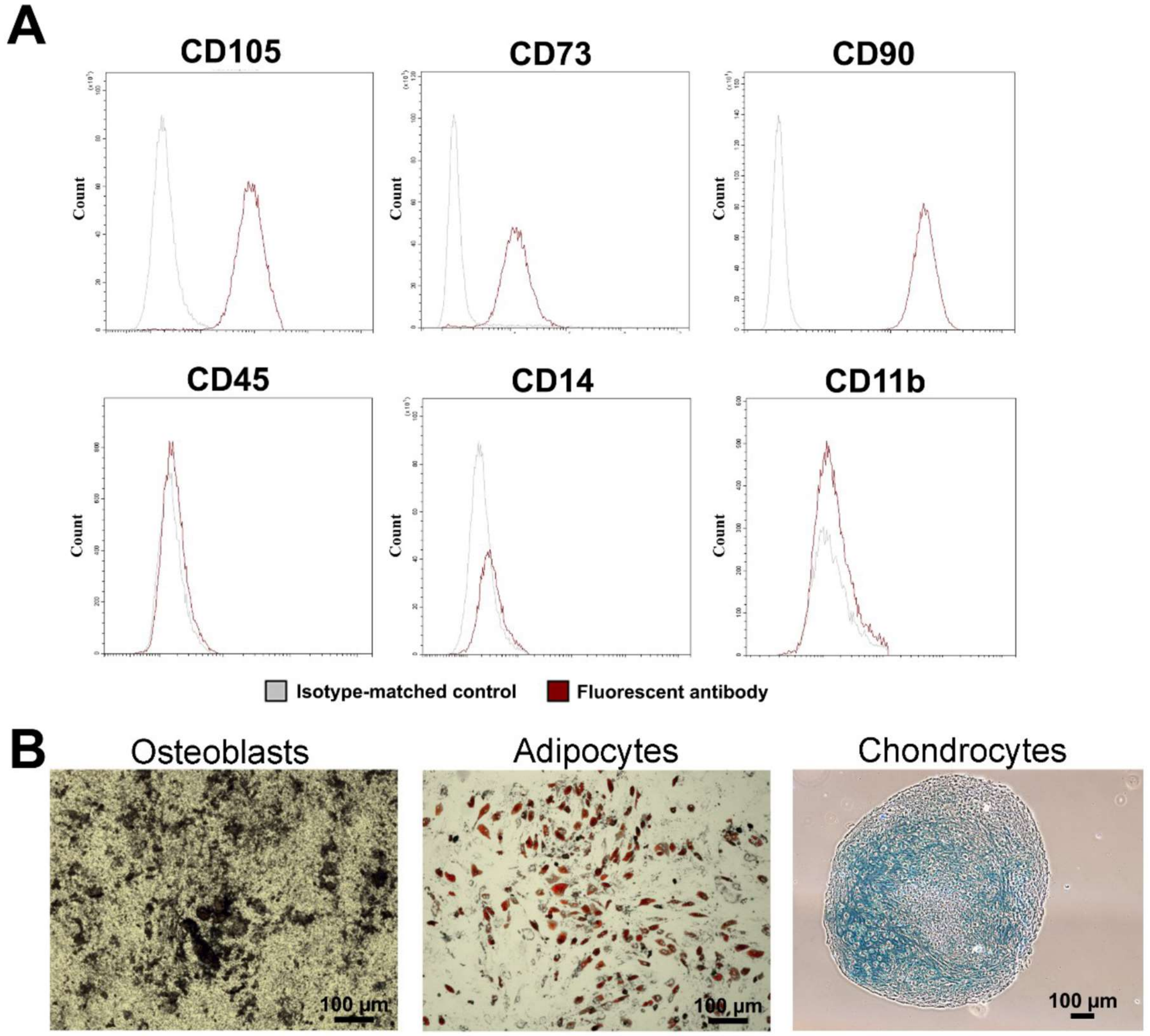

Figure S1. Morphological and functional characterization of human MSC. (A) Flow cytometry analysis of cell surface markers, illustrating that all cells were positive for CD105, CD90 and CD73 and negative for CD45, CD14 and CD11b. (B) Multipotency of MSC confirmed by specific histological stainings: von Kossa staining for osteoblasts, Oil Red O staining for adipocytes and Alcian blue staining for chondrocytes.

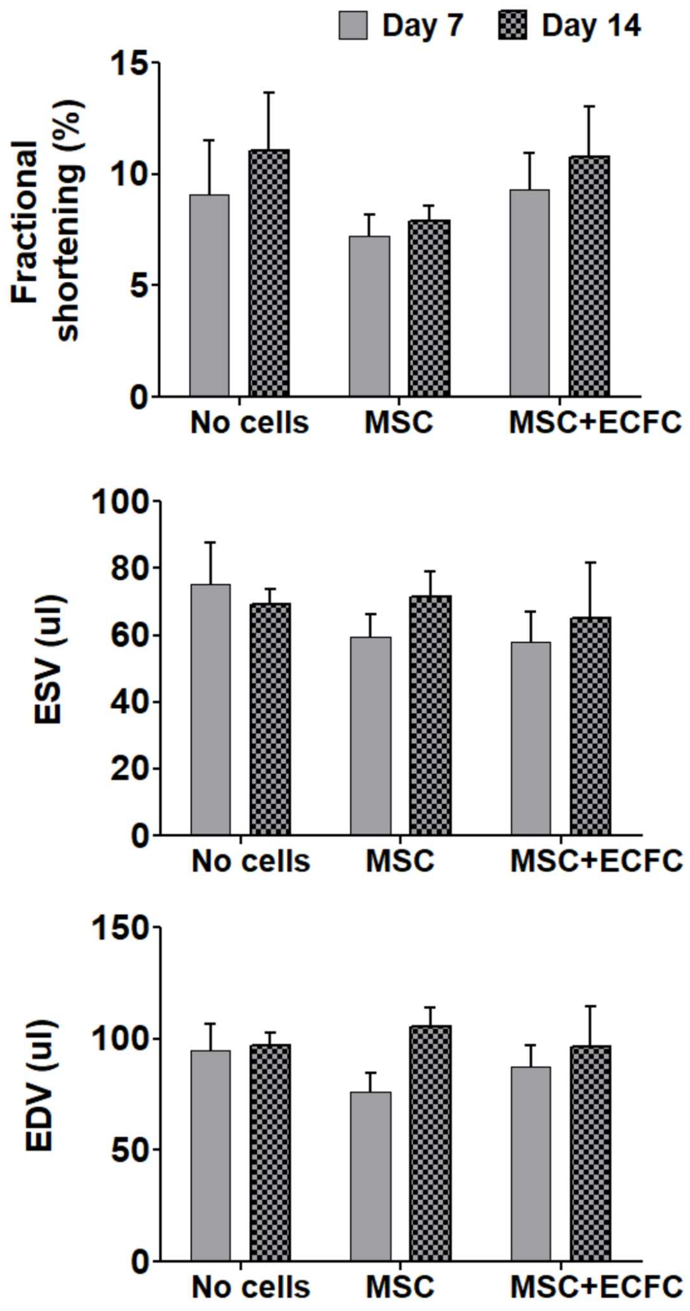

Figure S2. Echocardiographic measurements performed at Days 7 and 14 after surgery. No statistically significant changes were detected in terms of Fractional shortening, End Systolic Volume (ESV) or End Diastolic Volume (EDV).

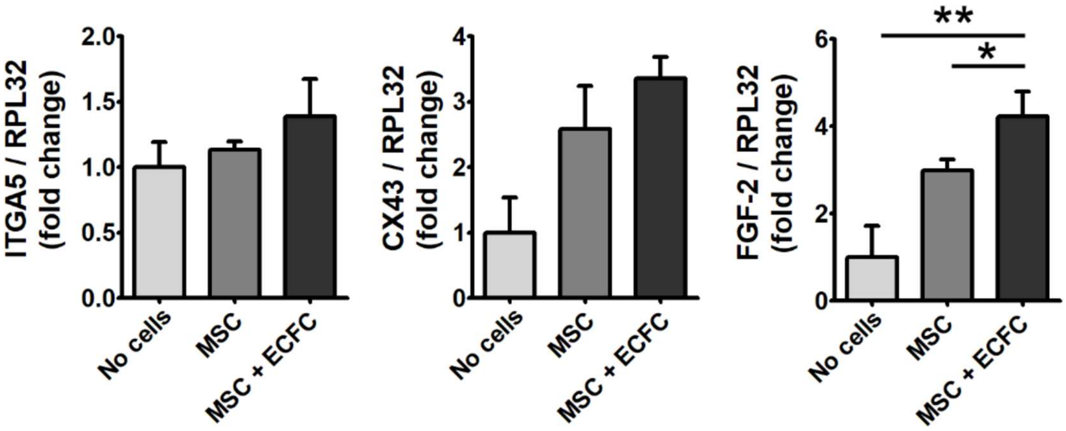

Figure S3. Changes in ITGA5, CX43, and FGF2 gene expression in mouse ventricles with myocardial infarction, evaluated at 14 days after surgery. (Statistics: One-way ANOVA followed by Tukey's multiple comparison test: * p value < 0.05; ** p value < 0.01).

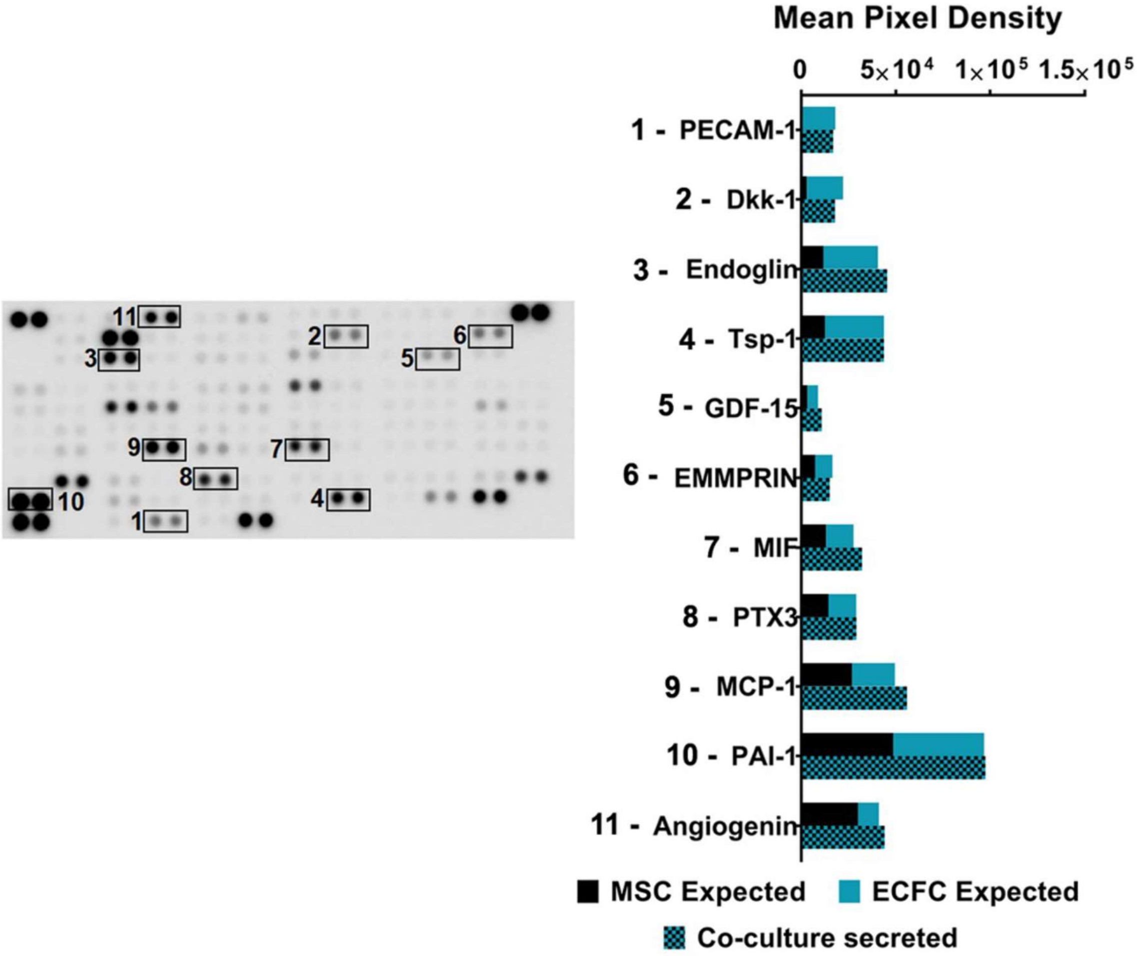

Figure S4. Proteomic analysis of the secretome produced by MSC and ECFC co-cultured in direct contact. The profiles of proteins secreted by MSC, ECFC and MSC+ECFC cultures were identified by antibody-based Human XL Cytokine Array. Black frames indicate highly secreted proteins whose expression in the co-culture sample are within the expected cumulated levels. Individual estimated contribution of MSC and ECFC to the levels of each cytokine, as compared to those found in the co-culture media, indicating consistency in their individual secretory activity after direct co-culture.

Table S1. Primer sequences.

**Gene**	***Forward Primer (5′ → 3′)***	***Reverse Primer (5′ → 3′)***
GAPDH	GCTCATTTCCTGGTATGACAACG	TCCTCTTGTGCTCTTGCTGG
RPL32	TCGTCAAAAAGAGGACCAAGAAG	CCGCCAGTTTCGCTTAATTT
ITGA5 (human)	ACCAAGGCAGAAGGCAGC	AGGGAGCGTTTGAAGAATCCAA
ITGA5 (mouse)	GCAGGATAGAGGACAAGGCCCA	TCATAGGCACCGCCCTCACC
FGF-2	AACTCCAAGCAGAAGAGAGAGG	CAGTGCCACATACCAACTGG
CX43	CTGGTGGTGTCCTTGGTGTCTC	GCTGTCGTCAGGGAAATCAAAC
